# Molecular identification of polymorphic transposable elements in populations of the invasive ant *Cardiocondyla obscurior*

**DOI:** 10.1093/biomethods/bpae050

**Published:** 2024-07-13

**Authors:** Esther van den Bos, Jürgen Gadau, Lukas Schrader

**Affiliations:** Institute for Evolution and Biodiversity, University of Münster, Hüfferstraße 1, Münster 48149, Germany; Institute for Evolution and Biodiversity, University of Münster, Hüfferstraße 1, Münster 48149, Germany; Institute for Evolution and Biodiversity, University of Münster, Hüfferstraße 1, Münster 48149, Germany

**Keywords:** Transposon display, transposable elements, *Cardiocondyla obscurior*, polymorphic, sequence-specific amplified polymorphisms

## Abstract

Transposable elements (TEs) are found in virtually every eukaryotic genome and are important for generating *de novo* genetic variation. However, outside of costly and time-consuming whole-genome sequencing approaches, the set of available methods to study TE polymorphisms in non-model species is very limited. The Transposon Display (TD) is a simple yet effective technique to characterize polymorphisms across samples by identifying amplified fragment length polymorphisms using primers targeting specific TE families. So far, this technique has almost exclusively been used in plants. Here, we present an optimized TD protocol for insect species with small genomes such as ants (ca. 200–600 Mb). We characterized TE polymorphisms between two distinct genetic lineages of the invasive ant *Cardiocondyla obscurior*, as well as between neighboring populations of the New World lineage. We found active LTR/Ty3 retrotransposons, that contributed to the genetic diversification of populations in this species.

## Introduction

Around 1950, Barbara McClintock discovered the first selfish genetic elements capable of moving around in the genome [1]. Now, over 70 years later, transposons are known from all three domains of life [2, 3]. Still, transposable elements (TEs) are often neglected in genomic analyses as they are proclaimed to be part of the “junk” DNA and their identification, classification, and detailed examination is tedious due to for example massive extensions or losses in individual lineages [4, 5].

TEs represent a diverse group of genetic elements [4, 5] and vary in size, structure, activity, replication mechanism, and transposition machinery [6]. As powerful mutagens TEs can be major contributors to evolutionary change [7]. They can cause different types of mutations, affecting gene expression and/or structure, and thus can have a strong impact on the host’s phenotype [8]. For the most part, TE insertions are neutral but can also be deleterious, for example, when they impair a host gene function or induce genomic instability, leading to genetic disorders and diseases [9]. Rarely, but not unheard of, TEs contribute to adaptive genetic innovations (reviewed by Schrader and Schmitz [10]).

Abundance and activity of TEs in the genome are modulated by evolutionary processes (e.g. positive, or negative selection), and genetic (recombination and mutation) and molecular (e.g. host defense) mechanisms. As a consequence, their distribution in the genome is not random [6, 11]. Due to the wide range of silencing mechanisms (such as zinc-finger proteins, small RNA-based silencing strategies, DNA-methylation, and chromatin modifications), spontaneous transposition events are usually rare [12–15]. However, a study on *Drosophila melanogaster* populations has shown that integrating the genetic variation across natural populations increased the number of detected TE insertions by more than 50% [16], emphasizing that TEs are an important source for genetic variation in natural populations. Detecting these TE polymorphisms, however, is still difficult, in particular with short-read DNA sequencing data [17].

The Transposon Display (TD) is a molecular technique that allows for rapid and specific screening of polymorphic TEs across individuals, populations, lineages, and species [18–21]. As a variation of the TD, the sequence-specific amplified polymorphism (SSAP) assay uses primers that recognize the flanking regions of a particular TE family and adjacent restriction sites in the flanking genomic DNA to amplify the region between them. Fragment sizes depend on the distances between transposon insertion sites and neighboring restriction cut sites. Consequently, as the resulting DNA amplicons are separated by gel electrophoresis, each insertion yields a band of distinct size, and differences in insertion sites between genomes manifest as noticeable variations in band patterns, generating a sample-specific fingerprint of TE polymorphisms [22, 23]. Although the TD is a relatively simple and cost-efficient technique, its high specificity delivers a focused view on individual TE families present in the genome. Thus, SSAP and related TD techniques are a valuable tool for studying the role of TEs in genome evolution.

In 2014, Schrader *et al*. found discrete transposon accumulations (TE-islands) in the otherwise TE-poor genome of the small, invasive ant *Cardiocondyla obscurior.* Inbreeding and repeated exposure to stress are suspected to have facilitated the emergence of these accumulations as a consequence of relaxed selection and low meiotic recombination. So despite high stochastic drift, TE islands may generate genetic novelty and, by chance, contribute to the emergence of adaptive phenotypes [11]. Populations of *C. obscurior* belong to two genetically and phenotypically distinct lineages [11, 24]: the Old World lineage is widespread (e.g. Japan, Taiwan, USA, Spain, and in greenhouses in Northern Europe), whereas the New World lineage has so far only been found in Brazil [25]. The genetic and phenotypic divergence of these lineages has been attributed partially to the activity of TEs within the last 40 000 generations [24]. Therefore, TEs have taken the spotlight as important contributors to genome structure, genetic diversification, and adaptation in *C. obscurior*.

In order to identify and study active TEs and their contribution to the genetic differentiation of colonies, populations, and lineages of *C. obscurior*, we modified the SSAP technique for the application in non-model species with small genomes and relatively low TE content. Applying the new protocol, we identified TE-dependent polymorphisms between geographically isolated populations of *C. obscurior*, consistent with recent activity of these TEs in the genome. We thereby demonstrate that our optimized TD protocol is a cost-effective and accessible method to assess TE polymorphisms in non-model insect species and provide further evidence that TEs contribute to genetic diversification in invasive populations of *C. obscurior*.

## Materials and methods

The invasive ant species *Cardiocondyla obscurior* belongs to the subfamily Myrmicinae. *C. obscurior* lab colonies, originally collected in Bahia, Brazil in 2018 and in a greenhouse in Freising, Germany in 2015 were kept in a climate chamber with a 12 h day/night schedule at 26°C/22°C and a humidity of 75%.

Five queen pupae of *C. obscurior* were sampled from colonies in liquid nitrogen for Cetrimonium Bromide (CTAB) DNA extraction following a modified method from Sambrook and Russel [26]. In brief, samples were crushed using small pestles before adding CTAB, proteinase k, chloroform, isoamyl, and sodium acetate. The combination of an enzyme, organic solvents, and salts increases the efficiency of protein denaturation and phase separation. After isolating the aqueous phase, isopropanol was added to precipitate the DNA. The sample was repeatedly washed with ethanol before resuspending the DNA in water.

**Figure 1.**
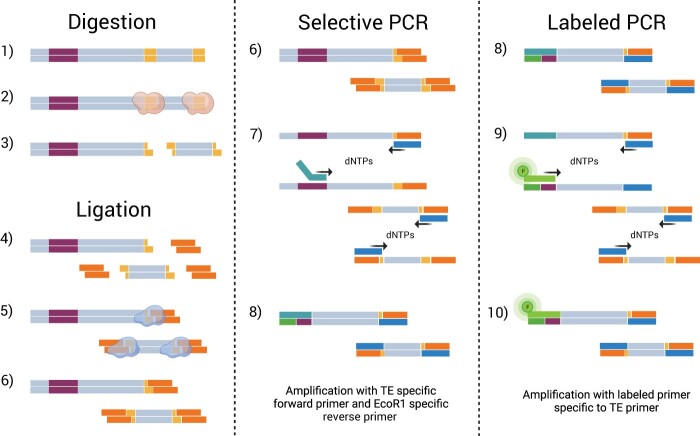
TD workflow. Digestion (1–3): genomic DNA (double stranded (ds) sequence) containing TE (violet ds sequence) is digested using a restriction enzyme (red cloud) with its specific restriction site (yellow ds sequence). Ligation (4–6): adaptor oligonucleotides (orange ds sequence) are ligated onto the digestion ends using a ligase (blue clouds). Selective PCR (6–8): a TE-specific forward primer (dark turquoise singlestranded (ss) sequence) and a restriction enzyme/adaptor-specific reverse primer (blue ss sequence) amplify sequences between TEs and restriction sites. The TE-specific forward primer is equipped with a 20 bp tail whose complementary sequence (green ss sequence) is annealed during PCR. Labeled PCR (8–10): using product of the first PCR, the amplicons between TE and restriction site are amplified using a fluorescently labeled forward primer (bright green ss sequence with fluorescence (F)) and the same enzyme/adaptor-specific reverse primer. Amplicons of interest are thereby fluorescently labeled. Without the fluorescent label amplicons between the reverse primers would also be visible in gels. Created with BioRender.com.

We adapted a SSAP method for the molecular characterization of candidate TEs and TE-dependent polymorphisms in *C. obscurior* (Fig. 1). To summarize, genomic DNA was digested using a restriction enzyme (**Digestion**), followed by the ligation of designed adaptors specific to the restriction enzyme’s recognition site (**Ligation**). In two PCR steps, genomic regions between LTR transposons and restrictions sites were amplified (**selective PCR**) and fluorescently labeled (**labeled PCR**). The PCR products were separated by gel electrophoresis and visualized under fluorescent light.

The use of a fluorescently labeled primer in a second round of PCR ensured that only amplicons spanning from the targeted transposon to a restriction site were stained, as opposed to amplicons between two restriction sites. Our design with a single universal fluorescent primer capable of binding to various TE-specific forward primers is more cost-effective than individually labeling each forward primer. It also allows for combining multiple TE-specific primers within a single TD, so that, for example, insertion polymorphisms of several different low-copy transposon families can be explored in the same assay.

### Transposon display protocol

#### Design and selection of adaptor oligonucleotides and primers

First, we designed double-stranded (ds) adaptors specific to the utilized restriction enzyme which were ligated to the cut ends of the restriction fragments. Adaptors entailed an enzyme-specific sequence complementary to the restriction enzyme’s recognition site followed by a random sequence that provided enough length for the annealing of PCR primers. We used the restriction enzyme *Eco*R1 and the specific adaptors: 5′-CTCGTAGACTGCGTACC-3′ and 5′-AATTGGTACGCAGTCTAC-3′.

Next, we designed a reverse primer used in both PCRs complementary to the adapter oligonucleotides, optimizing for a T_m_ between 55 and 60°C: 5′-GACTGCGTACCAATTC-3′.

The forward primers differ between selective and labeled PCR (Fig. 1). For the selective PCR, TE-specific forward PCR primers were designed to match an LTR sequence within 100 bp of its 3′-end with the primer oriented outwards, with 40%–60% GC-content, and T_m_ between 55 and 60°C. For primer design, *in-silico* PCRs were run to predict the expected number and size of amplicons. We only selected primers with 19–60 *in-silico* amplicons. The computational steps for designing and selecting primers are described in detail in Supplementary Material S1. In essence, they comprise: (i) identifying LTR retrotransposons using LTRharvest (alternatively, any available TE annotation for the study organism can be used), (ii) extracting terminal repeat motifs (e.g. LTR sequences) of an element of interest, (iii) extracting 20 bp oligonucleotides as potential primer sequences, and (iv) conducting in silico PCRs using individual primers and a restriction site (e.g. *Eco*R1) as reverse primer to estimate amplicon numbers and size (e.g. with in_silico_pcr, https://github.com/egonozer/in_silico_pcr). Optionally, the primer binding sites and expected amplicons can be visualized using a genome browser such as IGV (https://www.igv.org/).

For economic reasons, the designed TE-specific oligonucleotide primers were extended at the 5′-end to contain a recognition site for a fluorescently labeled primer, allowing us to pair inexpensive, regular TE-specific primers with the more expensive fluorescent-labeled primer in our experiments. The recognition site was designed as a random 20 bp sequence not found in the genome of the targeted organism so that fluorescent primers exclusively bind to amplicons from the first PCR (5′-TGTATACTGGAGCTGAGCTT-3′). The addition of 20 bp to the previously designed selective forward PCR primers increased their T_m_, which did not affect PCR performance.

The sequence of the fluorescent primer was complementary to the tail sequence of the TE-specific primers (see above). The fluorescent label of the primer can be chosen according to individual needs and preferences (i.e. capabilities of available imager, autofluorescence of utilized agarose). We tested three different labels, 6-Carboxyfluorescein (6-fam), Cyanine 5 (Cy5), and Cy3, of which Cy3 provided the best imaging results. Additional details regarding the designed primers can be found in Table 1.

**Table 1.** List of designed adaptors and primers for TD.

Primer	Type	Sequence	Length (bp)	T_m_ (°C)	No. Amp. (fw/rv)	Amp. size (min/max)	Exc./Em. (nm)
*Eco*R1 adaptors	5′–3′	CTCGTAGACTGCGTACC	17				
	3′–5 ′	CATCTGACGCATGGTTAA	18				
*Eco*R1 primer	rv	CTCGACTGCGTACCAATTC	19	55	–	–	–
CobsR.176: 1394-1413	fw	TGTATACTGGAGCTGAGCTTATTGAGTGTAGTTAGAGTAA	40	65	13/14	67/4799	–
LTR_retro450: 403-422	fw	TGTATACTGGAGCTGAGCTTATTGTAGATGTCGCTTGTGT	40	71	12/16	34/4993	–
LTR_retro798: 407-426	fw	TGTATACTGGAGCTGAGCTTGAAGATAGTGCGCGGCACTA	40	76	17/15	202/4802	–
LTR_retro170: 15/4,452,264	fw	TGTATACTGGAGCTGAGCTTTTTCGCCTAGGCAATGAC	38	74	13/21	178/4655	–
Cy3 labeled primer	fw	Cy3-TGTATACTGGAGCTGAGCTT	20	51	–	–	554/568

Table shows adaptor/primer name, primer type (forward/reverse), sequence, length, and melting temperature for all used primers. For TE-specific forward primers, number, and size of predicted amplicons from *in silico* PCRs with the *Eco*R1 reverse primer are included. For the labeled primer, wavelength of excitation and emission are included.

To assess primer suitability, *in-silico* PCRs were performed using the TE-specific forward primers and the *Eco*R1 reverse primer. The resulting number of amplicons (<5 kb) as well as predicted amplicon sizes were used to inform primer selection.

#### Genomic DNA digestion

For *EcoR1* digestion, we mixed 5 µl of Tango Buffer, 12.7 µl of sterile water, and 0.1 µl (1 U) of *Eco*R1, before adding 250 ng of genomic DNA in a volume of 7 µl (final volume 25 µl) and mixing gently. The digestion mix was incubated for 3 h at 37°C, followed by 15 min at 70°C to terminate the reaction.

##### Materials

- Tango Buffer (10×): 33 mM Tris-acetate (pH 7.9), 10 mM Mg-acetate, 66 mM K-acetate, 0.1 mg/ml BSA (10× Buffer Tango with BSA; Fermentas; cat#: BY5).
- Restriction enzyme (10 U/µl): *Eco*R1 is a rare cutter enzyme with the restriction site 5′-GAATTC-3′.

#### Ligation

After digestion, we added 3 µl of Tango Buffer, 5 µl of sterile water, 1 µl of ATP (20 µM), 1 µl *Eco*R1-adaptors (5 µM), and 5 µl (5 U) of T4 DNA Ligase to 25 µl of digestion mix (final volume 40 µl). The ligation reaction was incubated over night at RT. Afterward, we ran 5 µl of the mix on a 1% agarose gel electrophoresis to verify successful digestion. Ligation of the adaptor to the cut end of the DNA fragments replaced the restriction enzymes recognition site and thereby inhibited further restriction. Finally, we diluted the digestion–ligation mix 1:8 with sterile water. The diluted digestion–ligation mix can be stored at −20°C.

##### Materials

- *Eco*R1-adaptors (100 µM): 5′-CTCGTAGACTGCGTACC-3′ and 5′-AATTGGTACGCAGTCTAC-3′
  Preparation: we mixed equal volumes of the two adaptors (50 µM), incubated at 95°C for 5 min, and diluted them 1:10 (5 µM).
- ATP (20 µM; ATP Solution; Invitrogen by Thermo Fisher Scientific; MAXIscript 100 rxns Kit; 100 µl, 10 mM; REF: 8110G)
- T4 DNA ligase (5 U/µl; T4 DNA Ligase; Thermo Scientific; 1 Weiss u/µl, 500 u, 200 CEU/µl; cat#: EL0016)

#### PCR for selective amplification

To 2 µl of the diluted digestion-ligation mix we added 2 µl of Rxn Buffer, 10.4 µl of sterile water, 2.5 µl dNTPs (10 mM), 1.6 µl MgCl_2_ (25 mM), 0.8 µl of tailed TE-specific forward primer (20 µM), 0.5 µl of *Eco*R1 reverse primer (20 µM), and 0.2 µl *Taq* polymerase (5 U/µl). PCR amplification was done in a thermocycler as follows:

**Table:** 

1×	180 s	94°C
8×	30 s	94°C
	60 s	62.5–58°C (−1.5°C every second cycle)
	240 s	72°C
20×	30 s	94°C
	60 s	56.5°C
	240 s	72°C
1×	180 s	72°C
Hold		4°C

After PCR, we diluted the selective amplification product 1:20 with sterile water.

##### Materials

- TE-specific forward primer with tail (20 µM)
- *Eco*R1 reverse primer (20 µM)
- Rxn Buffer (10×): 200 mM Tris pH 8.4, 500 mM KCl
- dNTPs (10 mM)
- MgCl_2_ (25 mM)
- *Taq* polymerase (5 U/µl; GoTaq G2 Flexi DNA Polymerase; 500 u; REF: M7808)

#### PCR for labeled amplification

To 3 µl of the diluted selection amplification product we added 2 µl of Rxn Buffer, 9.4 µl of sterile water, 2.5 µl of dNTPs (10 mM), 1.6 µl of MgCl_2_ (25 mM), 0.8 µl of labeled forward primer (20 µM), 0.5 µl of *Eco*R1 reverse primer (20 µM), and 0.2 µl of *Taq* polymerase (5 U/µl). The PCR was performed in a thermocycler as follows:

**Table:** 

1×	120 s	94°C
8×	30 s	94°C
	30 s	62.5–58°C (−1.5°C every second cycle)
	240 s	72°C
20×	30 s	94°C
	30 s	56.5°C
	240 s	72°C
1×	300 s	72°C
Hold		4°C

##### Materials

- Labeled forward primer (20 µM)
- *Eco*R1 reverse primer (20 µM)
- Rxn Buffer (10x)
- Equimolar dNTPs (10 mM)
- MgCl_2_ (25 mM)
- *Taq* polymerase

#### Electrophoresis

We adjusted the electrophoresis settings to the predicted band sizes of the amplification products and to the application of a fluorescently labeled primer by increasing the agarose percentage and avoiding usage of any stains or dyes (e.g. Roti gel stain, green loading buffer). Only GelStar (1000×) was added into the gel pockets of the DNA ladders.

We predominantly prepared 2% agarose gels with TAE buffer for sharper bands. We loaded the PCR product of the second (labeled amplification) PCR on the gel and ran the electrophoresis for 4 h at 60 V (BIO RAD Power Pac 300). We covered the chamber with aluminum foil during the run to preserve fluorescence.

After the run we imaged the gel (Alpha Innotech, FluorChem Q, Biozym, MultiImage III) using the appropriate settings of the utilized label.

#### Sequencing of gel bands

While the electrophoresis of labeled PCR products reveals TE-specific amplicons, the fluorescent label used interferes with sequencing. Thus, for extracting TE-specific gel bands for Sanger sequencing, we ran parallel gel electrophoreses on the selective and labeled PCR products. By aligning and comparing both gels, we could then identify and extract TE-specific bands in the selective PCR gel (stained with GelStar) based on the position of corresponding fluorescent bands in the labeled PCR gel. Extracted bands were processed with the Zymoclean Gel DNA Recovery Kit (Zymo Research; 200 preps.; cat#: D4008) and the DNA Clean & Concentrator-25 Kit (Zymo Research; 200 preps.; cat#: D4034) prior to sending the samples for Sanger sequencing. The DNA output (measured with the IMPLEN P330 Flame Nanophotometer) ranged between 25 and 50 ng/µl.

## Results

We designed this TD protocol to target SSAPs of LTR retrotransposons across lineages, populations, and colonies of *Cardiocondyla obscurior*.

We conducted TDs targeting four different LTR retrotransposons using specific primers. In each TD, we compared TE polymorphisms using TD band patterns across four samples of *C. obscurior* collected from three different populations. One sample was collected from colonies retrieved from a greenhouse in Freising, Germany (referred to as F) belonging to the Old World lineage [11]. For the New World lineage one sample was collected from a population in Itabuna, Brazil (sample I), and two samples from another Brazilian population approximately 50 km away in Una (samples U1, U2).

**Figure 2.**
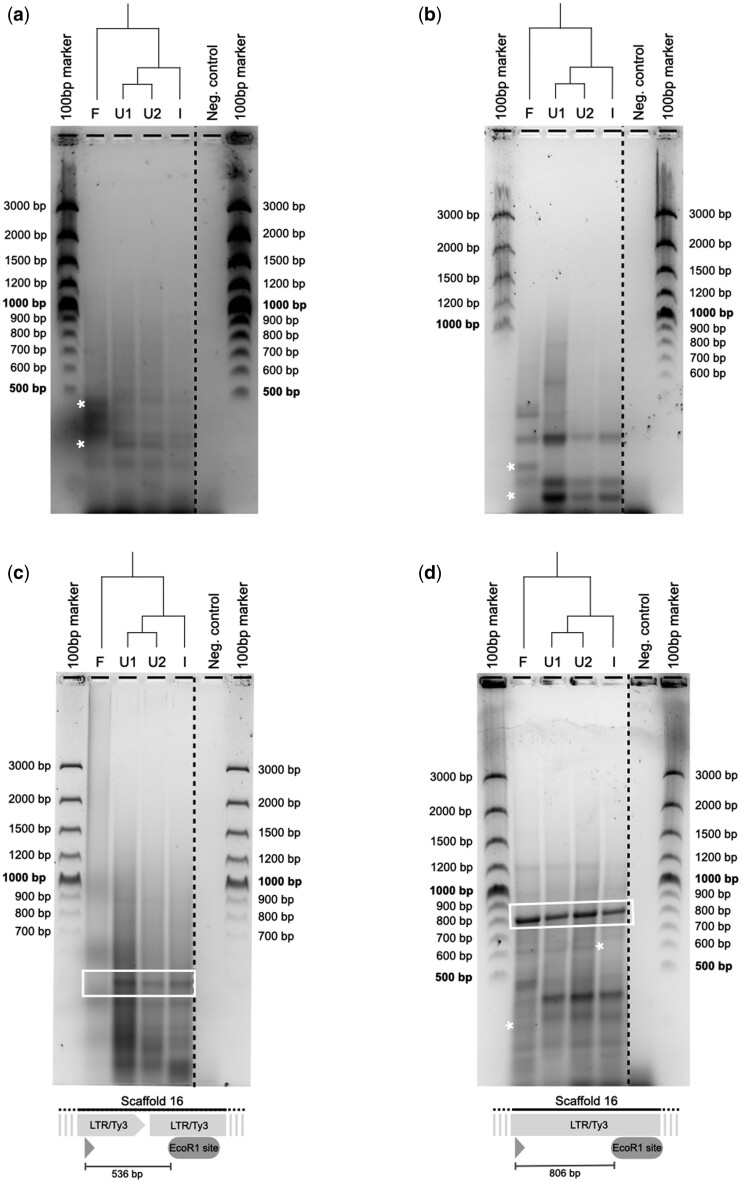
TDs targeting the LTR/Ty3 families CobsR.176 (**a**), LTR_retro798 (**b**), LTR_retro450 (**c**), and LTR_retro170 (**d**). Gel lanes contain (1) a 100 bp marker, samples from (2) Freising (DE), (3) Una 1 (BR), (4) Una 2 (BR), (5) Itabuna (BR), (6) a negative control (water was added to the reaction mix instead of DNA and then run through all transposon display steps), and (7) another 100 bp marker. White asterisks mark band polymorphisms. White box mark bands were excised and sequenced. Striped, black lines indicate position of spliced out lanes. Alignments of sequenced bands against the annotated genome of *C. obscurior* are visualized in panels (c) and (d).

The first retrotransposon we targeted was CobsR.176 (Table 1), a putatively active LTR/Ty3 family with at least 16 full-length copies in the genome of *C. obscurior* according to a population genomic screening [24]. We designed a primer targeting the internal region of the element, downstream of the flanking long-terminal repeats of this retrotransposon. The TD produced several clearly discernable bands in each of the four tested samples (Fig. 2a). We also found at least three differences when we compared band patterns between the German (F) and Brazilian (U1, U2, I) samples. There were no differences between the Brazilian samples. Similarly, TDs targeting LTR/Ty3 families LTR_retro798 (Fig. 2b) and LTR_retro170 (Fig. 2d) revealed several TE-dependent polymorphisms between the German and the Brazilian populations. The TD targeting LTR_retro170 further revealed one difference between the Itabuna (I) and the Una (U1 and U2) and Freising (F) populations at 600 bp.

Two high intensity gel bands were extracted (white boxes in Fig. 2c and d) and Sanger sequenced to control for correct amplification of LTR/Ty3 loci. Sequences of extracted bands were aligned to the *C. obscurior* genome using Geneious^®^. Both sequences fit the predicted amplicon of the utilized primer pairs and map to the targeted LTR/Ty3 elements. Predicted amplicon lengths of the alignment for LTR_retro450 (Fig. 2c) at 536 bp and LTR_retro170 (Fig. 2d) at 806 bp coincide with the associated gel images and band lengths affirming the specificity of our TE-specific primers.

Sequencing and alignment of the excised band of the TD targeting LTR_retro450 showed that it is amplified from a genomic locus where two LTR/Ty3 elements occur in tandem on Scaffold 16. The sequence of the excised band of TD LTR_retro170 aligns entirely within the TE element, showing that both primer binding site and *Eco*R1 recognition site occur within the TE. Thus, any full-length copy of the element provides a template in the PCR, likely explaining the high intensity of this band.

To confirm that the Cy3-labeled forward primer alone does not produce non-specific amplicons in the labeled amplification, we repeated our assay, but including reactions lacking *Eco*R1 reverse primers as additional negative controls. We did not find any amplicons in these negative controls (Supplementary Material S2).

## Discussion

The TD protocol described in this study was adapted from the SSAP method for the molecular characterization of candidate TEs and TE-dependent polymorphisms in the invasive ant species, *Cardiocondyla obscurior*. The method involves several steps, including DNA extraction, digestion, ligation, selective PCR amplification, labeled PCR amplification, gel electrophoresis, and sequencing of gel bands for validation of key candidates. Each step was carefully designed to maximize the specificity and reliability of the assay.

A critical aspect of the TD protocol is the design of primers and adaptors tailored to the characteristics of the target species and TEs. The adaptors, specific to the utilized restriction enzyme (*EcoR1*), are ligated to the cut ends of the restriction fragments, replacing the restriction enzyme recognition site, and inhibiting further restriction. The TE-specific forward primers, designed to match the LTR sequences within 100 bp of their 3′-end, are optimized for GC-content and annealing temperature to ensure specific amplification. The addition of a fluorescent label to the forward primer in a second PCR allows for the visualization of amplicons derived from transposon insertion sites, enhancing the sensitivity and specificity of the assay. With the computational pipeline we provide here, primer design can be accomplished in the matter of minutes, even for researchers with little experience in bioinformatics.

We carefully optimized PCR conditions to ensure robust and specific amplification of transposon insertion sites. Parameters such as annealing temperature, cycling parameters, and reaction components were systematically explored and adjusted to maximize amplification efficiency while minimizing non-specific amplification. We also recommend performing *in-silico* PCRs to predict the expected number and size of amplicons, guiding primer selection and assay optimization.

Gel electrophoresis settings were tailored to the predicted band sizes of the amplification products and the application of a fluorescently labeled primer. The use of GelStar dye allows for the visualization of TE-specific bands in the selective PCR gel. Parallel gel electrophoreses of selective and labeled PCR products facilitate the extraction of TE-specific bands for Sanger sequencing. This approach overcomes the interference caused by the fluorescent label, ensuring accurate sequencing of transposon insertion sites.

The TD technique reliably works and has revealed several polymorphisms using our designed TE-specific forward primers in *C. obscurior*. Therefore, for studies aiming to examine the activity and transposition of a specific TE family, the TD provides an easy, robust, and inexpensive method to analyze TE-associated polymorphisms. Conducting TDs with the designed primers allowed us to uncover TE polymorphisms between Brazilian and German populations for all four of our targeted LTR/Ty3 elements (Fig. 2a–d). The prominent differences are consistent with the previously reported high genetic divergence of German and Brazilian populations of *C. obscurior* found in genomic studies [11, 24].

The band patterns across samples revealed by gel electrophoresis provide a visual comparison of TE patterns similar to a paternity test. The polymorphic nature of gel signals observed in our TD can be attributed to the presence of multiple copies of the targeted TE with variations in their flanking sequences, including the *Eco*R1 sites. Transposons, by their very nature, are mobile, capable of moving around in the genome and inserting themselves at various locations. In the case of our study on the ant genome, it is likely that there are multiple copies of a targeted transposon dispersed throughout the genome, each inserted at different genomic loci. Over time, the TEs themselves mutate. The presence of related TEs with similar sequences could contribute to the variability in gel signals as well. In addition, ant genomes, like many other eukaryotic genomes are complex and dynamic, with varying degrees of conservation among TEs and their flanking sequences. While certain transposon copies may exhibit high conservation, others may undergo genomic rearrangements, mutations, or amplifications, leading to differences in *Eco*R1 sites or adjacent sequences. Essentially, the polymorphic gel signals reflect the genomic diversity and complexity of TE distribution within the genome.

The patterning between Brazilian samples (U1, U2, I) is highly conserved for most of our targeted TEs. Solely LTR_retro170 (Fig. 2d) showed divergent amplicons in the TD between Brazilian populations, with one amplicon absent in Itabuna and present in the three other populations. The four targeted LTRs belong to LTR families with several complete copies in the genome of *C. obscurior*, based on structural annotations produced by LTRharvest, and are thus most likely to be currently active. The polymorphic insertions of these TEs revealed by our study can be explained by recent activity of the selected LTR/Ty3 families but also by ancient transpositions, preceding the split of the populations and further studies are required to resolve this uncertainty. Regardless, by confirming that polymorphisms associated with the four different families are segregating in populations of *C. obscurior*, our study encourages further focusing on CobsR.176, LTR_retro798, LTR_retro450, and LTR_retro170 as candidates for actively transposing TEs in the genome of *C. obscurior*. Identification of putatively active TEs will be valuable for future studies exploring how the unlikely genomic architecture of *C. obscurior* has evolved and is maintained, allowing for precise molecular experiments, without requiring labor-intense and expensive genome-wide sequencing efforts.

Overall, the number of amplicons produced in TDs in *C. obscurior*, which has the smallest genome known in ants [24], bode well for applications of our protocol in insects with small or medium sized genomes. The TD method was previously almost exclusively applied to study plant species although it offers the opportunity to study candidate TEs in a wide range of species. Active TEs are particularly interesting, because they generate novel genomic diversity, but identifying such TEs and their current activity is still difficult, even for typical model organisms [4, 27, 28]. A study on fire ants applied RNA profiling and genomic characterization methods, time-intensive and costly methods, to study the role of a specific element in the invasiveness of the species [29]. With our TD approach these types of contemporary studies become easier and more affordable, thereby providing new opportunities for studying TEs as major contributors to genomic and phenotypic diversity and adaptation in non-model insect species. By carefully designing primers and adaptors, optimizing PCR conditions, and employing adapted electrophoreses strategies, this inexpensive methodology presented here can serve as a valuable tool for researchers studying transposon biology in diverse organisms and experimental settings providing valuable insights into the dynamics of TEs and their contribution to genome evolution.

## Supplementary Material

bpae050_Supplementary_Data

## Data Availability

The genome assembly and raw sequencing data prior to trimming and mapping are available at NCBI (BioProject: PRJNA680013). A detailed description of our bioinformatic approach can be found in the Supplementary Material S1.
