# Supplementary material for: Molecular identification of polymorphic transposable elements in populations of the invasive ant Cardiocondyla obscurior
Source: Biol Methods Protoc. 2024 Jul 13;9(1):bpae050. doi: 10.1093/biomethods/bpae050 (PMC11268152; doi:10.1093/biomethods/bpae050)
Supplement: bpae050_Supplementary_Data [file bpae050_supplementary_data.zip › Gelimages with negative control for Cy3 labeled forward primer.pdf]

Gelimages with negative control for Cy3 fluorescently labeled forward primer

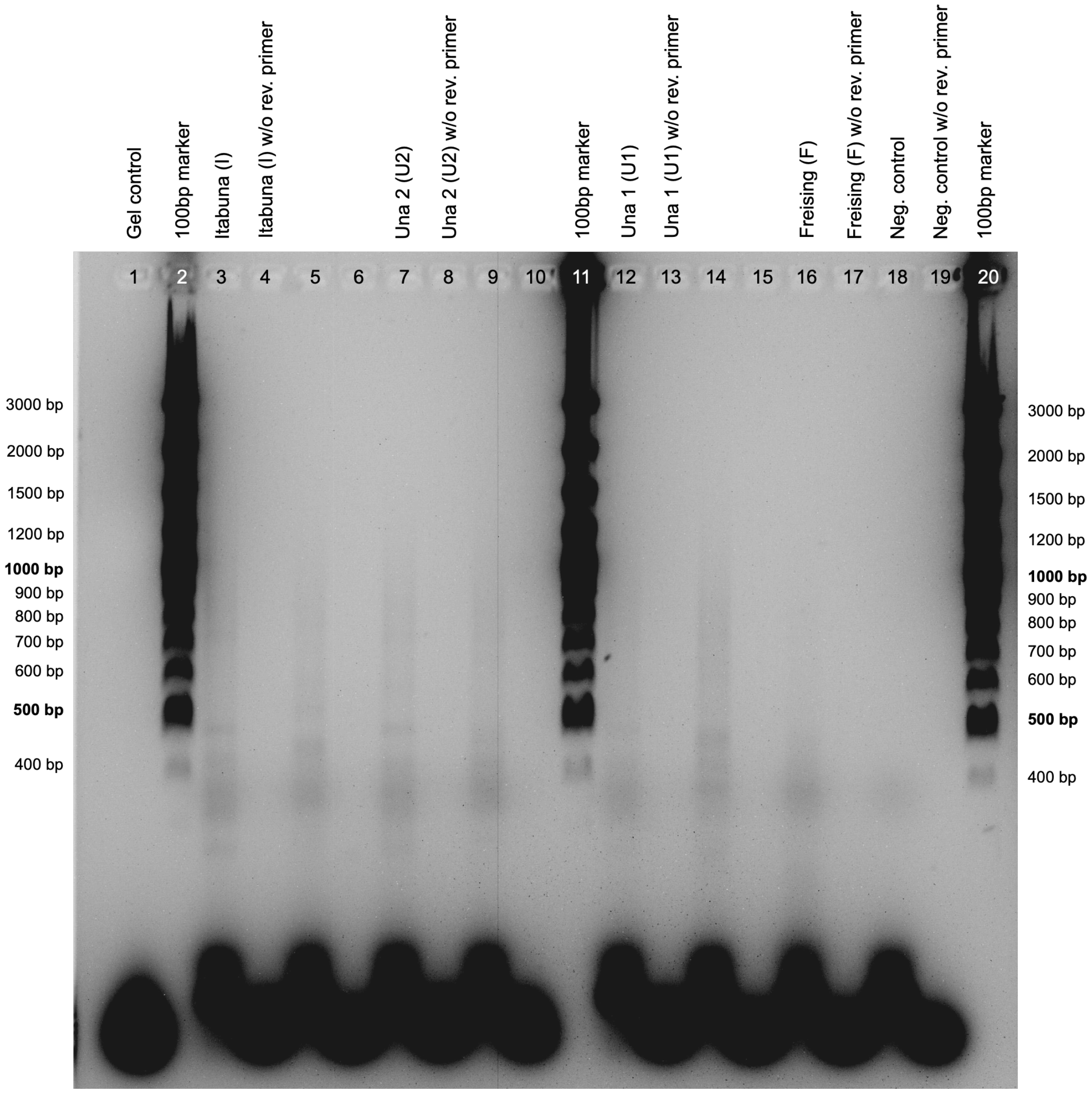

Supplementary Figure S1: Assay confirming no amplification in absence of EcoR1 reverse primers for TE-specific primer CobsR.176: 1394-1413. *Lane 1: gel control (loading buffer and labeled primer), lanes 2, 11, 20: 100 bp marker, lanes 3, 5, 7, 9, 12, 14, 16: complete TD reactions of different* Cardiocondyla *samples, lanes 4, 6, 8, 10, 13, 15, 17: TD reactions lacking* EcoR1 *reverse primers (negative control regarding non-specific Cy3-labeled primer amplification), lane 18: no template negative control (water was added to the reaction mix instead of DNA and then run through all transposon display steps), lane 19: no template, no reverse primer negative control . Lanes 3, 7, 12, 16: samples used in publication with lane description.*

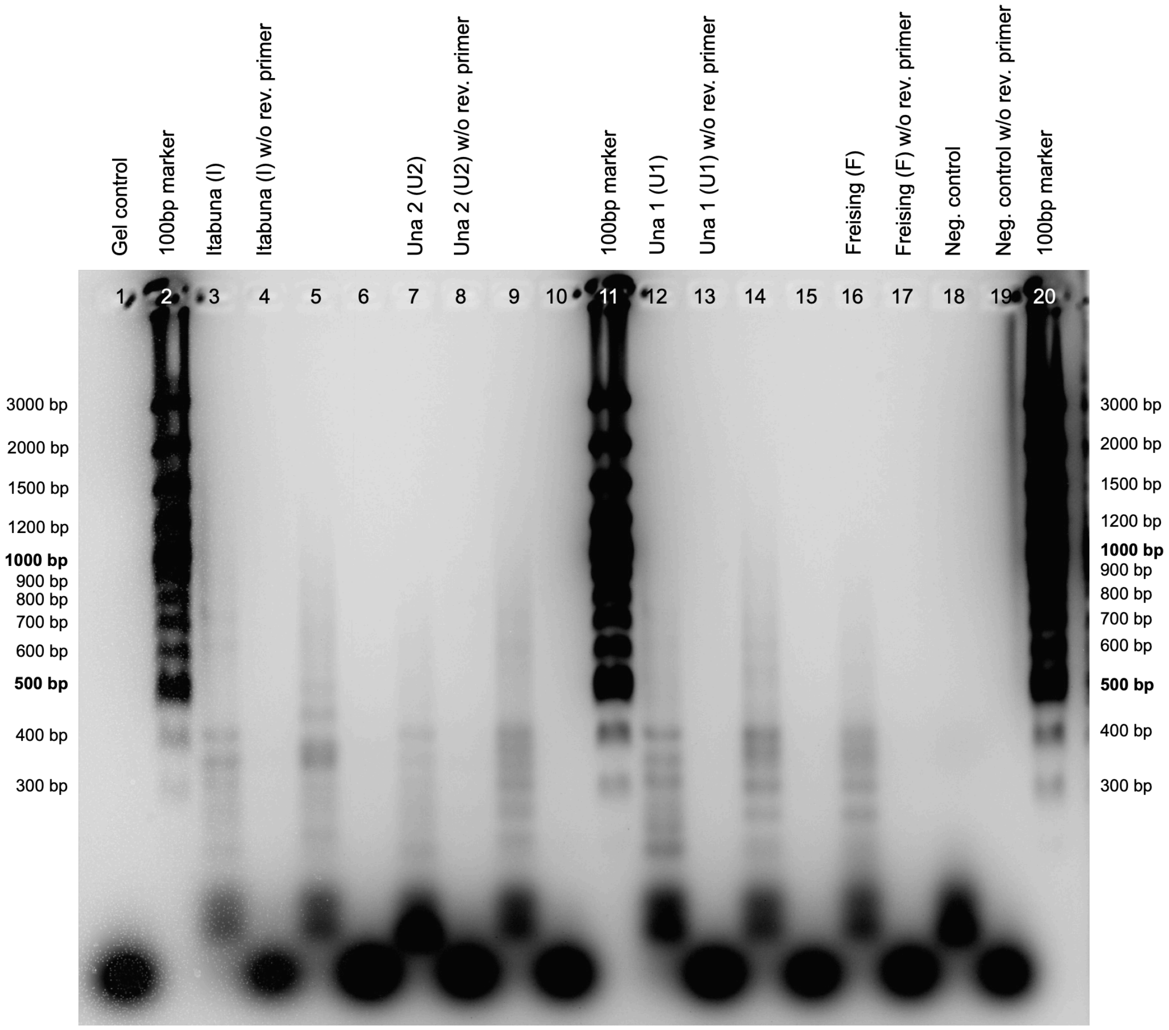

Supplementary Figure S2: Assay confirming no amplification in absence of EcoR1 reverse primers for TE-specific primer LTR_retro450: 403-422. *Lane 1: gel control (loading buffer and labeled primer), lanes 2, 11, 20: 100 bp marker, lanes 3, 5, 7, 9, 12, 14, 16: complete TD reactions of different* Cardiocondyla *samples, lanes 4, 6, 8, 10, 13, 15, 17: TD reactions lacking* EcoR1 *reverse primers (negative control regarding non-specific Cy3-labeled primer amplification), lane 18: no template negative control (water was added to the reaction mix instead of DNA and then run through all transposon display steps), lane 19: no template, no reverse primer negative control. Lanes 3, 7, 12, 16: samples used in publication with lane description.*

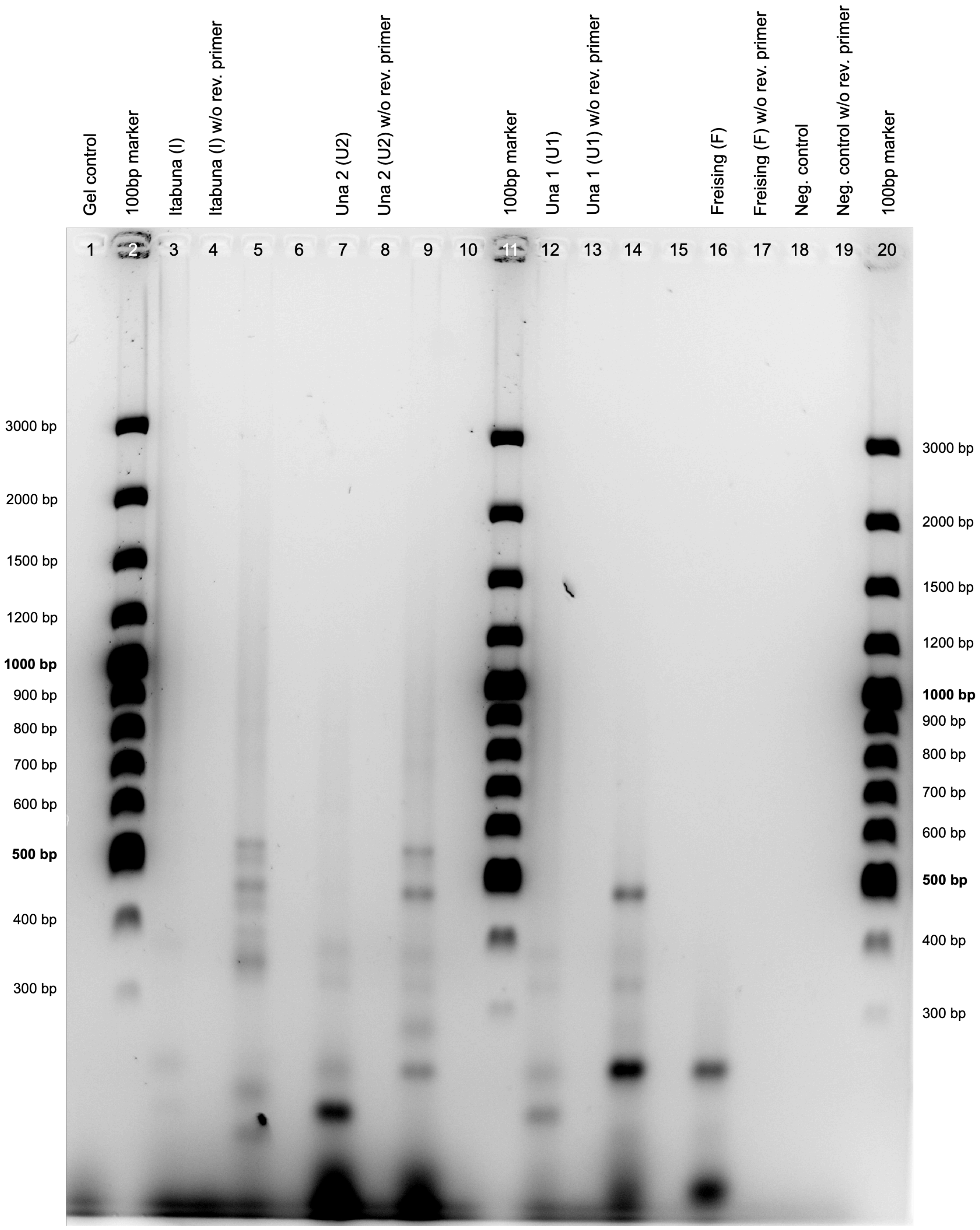

Supplementary Figure S3: Assay confirming no amplification in absence of EcoR1 reverse primers for TE-specific primer LTR_retro798: 407-426. *Lane 1: gel control (loading buffer and labeled primer), lanes 2, 11, 20: 100 bp marker, lanes 3, 5, 7, 9, 12, 14, 16: complete TD reactions of different* Cardiocondyla *samples, lanes 4, 6, 8, 10, 13, 15, 17: TD reactions lacking* EcoR1 *reverse primers (negative control regarding non-specific Cy3-labeled primer amplification), lane 18: no template negative control (water was added to the reaction mix instead of DNA and then run through all transposon display steps), lane 19: no template, no reverse primer negative control. Lanes 3, 7, 12, 16: samples used in publication with lane description.*

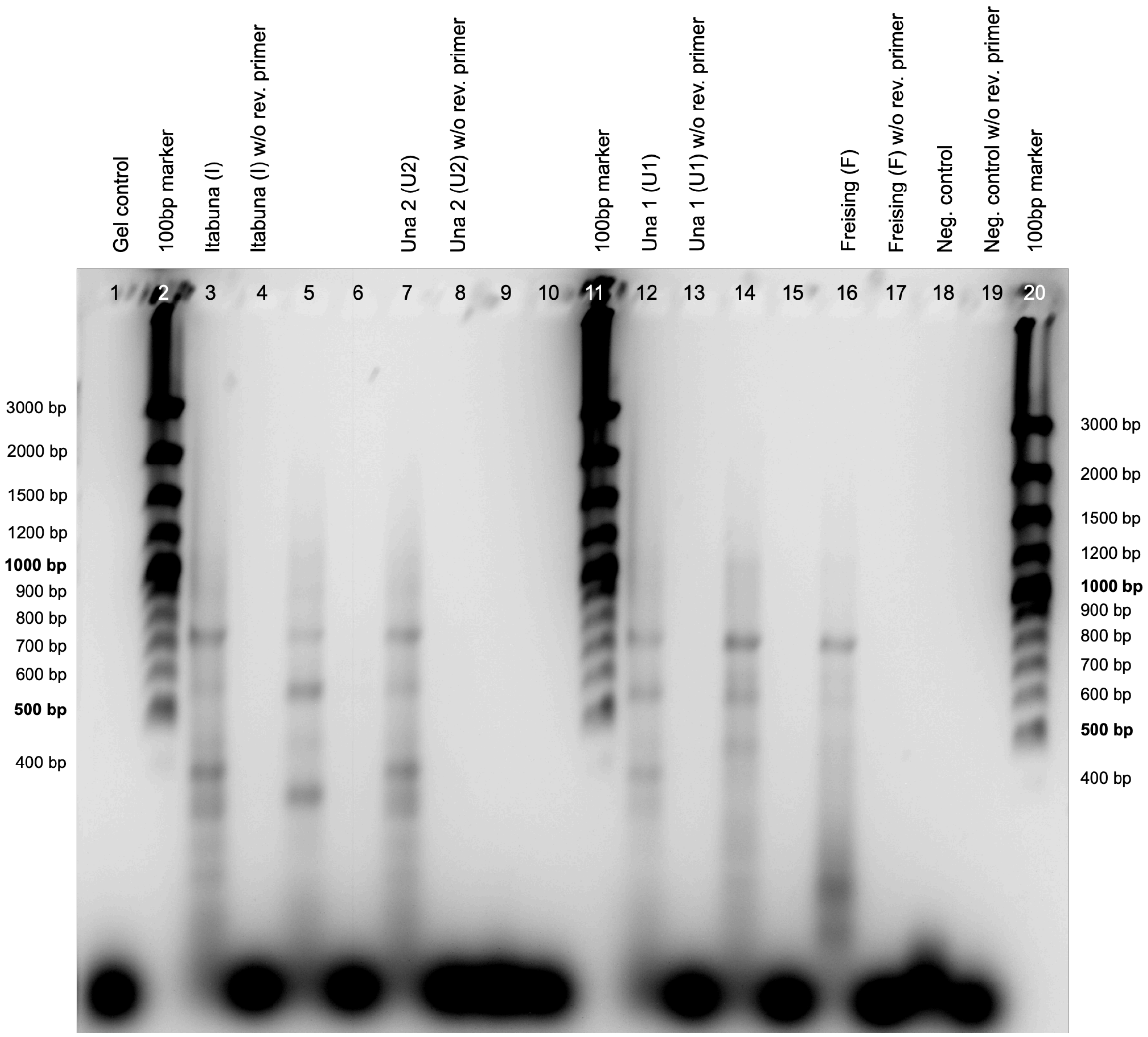

Supplementary Figure S4: Assay confirming no amplification in absence of EcoR1 reverse primers for TE-specific primer LTR_retro170: 15/4,452,264. *Lane 1: gel control (loading buffer and labeled primer), lanes 2, 11, 20: 100 bp marker, lanes 3, 5, 7, 9, 12, 14, 16: complete TD reactions of different* Cardiocondyla *samples, lanes 4, 6, 8, 10, 13, 15, 17: TD reactions lacking* EcoR1 *reverse primers (negative control regarding non-specific Cy3-labeled primer amplification), lane 18: no template negative control (water was added to the reaction mix instead of DNA and then run through all transposon display steps), lane 19: no template, no reverse primer negative control. Lanes 3, 7, 12, 16: samples used in publication with lane description.*
