# Supplementary material for: Molecular identification of polymorphic transposable elements in populations of the invasive ant Cardiocondyla obscurior
Source: Biol Methods Protoc. 2024 Jul 13;9(1):bpae050. doi: 10.1093/biomethods/bpae050 (PMC11268152; doi:10.1093/biomethods/bpae050)

Primer design for Transposon Display assays in species with compact genomes

This document is provided as Supplementary Material to the article *"Molecular identification of polymorphic transposable elements in populations of the invasive ant Cardiocondyla obscurior"* by Esther van den Bos, Jürgen Gadau, and Lukas Schrader.

Here, we describe the steps required to generate primers suitable for Transposon Display assays in species with compact genomes such as ants, bees, or other Hymenoptera. In this example, we design primers for LTR/Ty3 retrotransposons present in the genome of the invasive ant *Cardiocondyla obscurior*. The following code will produce primers that target the element `LTR_retrotransposon798` of the LTR/Ty3 family, as predicted by `LTRharvest` (see below). `LTR_retrotransposon798` is annotated as a representative of `ltrfam_8`, which is the most abundant LTRfam in the genome of *Cardiocondyla obscurior* according to `LTRharvest`.

Software Requirements

Software	Source	Purpose
LTR harvest	github.com/genometools/genometools	Annotation of LTR elements in the target genome
bedtools	github.com/arq5x/bedtools2	Fasta extraction of long-terminal repeats (LTRs)
mafft	mafft.cbrc.jp/alignment/software/	Alignment of LTRs
emboss cons	emboss.sourceforge.net/apps/cvs/emboss/apps/	Consensus calling for aligned LTRs
samtools	samtools.github.io	Fasta extraction of putative primer sequences ("dummy primers")
blast+	github.com/ncbi/blast_plus_docs	`blastn` searches of dummy primers against reference genome
in_silico_pcr	github.com/egonozer/in_silico_pcr	*in silico* PCRs
Tm calculator [R]	github.com/JunhuiLi1017/TmCalculator	Calculating Tm and GC of primers
Integrative Genomics Viewer (IGV)	software.broadinstitute.org/software/igv/	Visualization of primer binding sites

Run LTRharvest

Using LTRharvest, we annotate LTR elements in the genome assembly of *C. obscurior* (version 2.1) contained in the file Cobs.alpha.2.1.fa. In principle, any software to identify and annotate transposable elements (TEs) in the target genome can be used here. The Transposon Display will work best when targeting TEs present with several full-length copies in the genome.

LTRharvest is a software included in the genometools package that uses structural features of LTR elements for de novo annotation of such elements in the target genome.

In this example, we will annotate in the genome fasta file Cobs.alpha.2.1.fa, which contains the assembled reference genome for *Cardiocondyla obscurior*.

```
# Run LTRharvest for de novo annotation of LTR elements
## Create suffix array for genome fasta (required for subsequent annotation
with LTRharvest)
gt suffixerator -suf -lcp -tis -des -dna -ssp -db Cobs.alpha.2.1.fa -
indexname Cobs.alpha.2.1.fa
## Run LTRharvest to predict LTR retrotransposons
gt ltrharvest -index Cobs.alpha.2.1.fa -seqids -tabout no >
Cobs.alpha.2.1.ltrs.gff3
## Sort produced GFF file
gt gff3 -sort Cobs.alpha.2.1.ltrs.gff3 > Cobs.alpha.2.1.ltrs_sorted.gff3
## Identify and annotate LTR retrotransposons
### the GyDB_collection is available here:
https://gydb.org/extensions/Collection/collection/db/GyDB_collection.zip
gt ltrdigest -hmms GyDB_collection/profiles/*hmm -aaout -outfileprefix
ltrs_sorted -seqfile Cobs.alpha.2.1.fa -matchdescstart <
Cobs.alpha.2.1.ltrs_sorted.gff3 > Cobs.alpha.2.1.ltrs.digested.gff3


# Use gt select to filter full domain set (see
https://github.com/satta/filterama)
gt select -rule_files filter_full_domain_set.lua -- <
Cobs.alpha.2.1.ltrs.digested.gff3 > Cobs.alpha.2.1.ltrdigest.filtered.gff3

# Rerun ltrharvest to subsequently run gt ltrclustering
gt ltrharvest -index Cobs.alpha.2.1.fa -tabout no >
Cobs.alpha.2.1.ltrs.legacy.gff3
gt ltrclustering -psmall 80 -plarge 30 -o lologre_ltrclustering.out -seqfile
Cobs.alpha.2.1.fa lologre_ltrdigest.gff3

# Combine output from ltrclustering with ltrharvest
## extract sequence identifiers from Cobs.alpha.2.1.ltrs.digested.gff3
cat Cobs.alpha.2.1.ltrs.digested.gff3  |grep "##sequence-region"|sort -k 4,4
-nr > scf.seq.tsv
## extract sequence identifiers from lologre_ltrclustering.out
cat lologre_ltrclustering.out  |grep "##sequence-region"|sort -k 4,4 -nr >
```

```
legacy.seq.tsv
## create conversion table to replace identifiers in
lologre_ltrclustering.out
paste scf.seq.tsv legacy.seq.tsv|perl -pe 's/ +/\t/g'|cut -f 2,6 >
conversionTable.tsv
## create a converted gff with updated sequence identifiers
awk 'FNR==NR{a[$2]=$1;next}{print a[$1]"\t"$0}' conversionTable.tsv
lologre_ltrclustering.out|cut -f 1,3-11|awk 'NF' >
Cobs.alpha.2.1.ltrs.clustered.gff3
## Extract gff header
cat Cobs.alpha.2.1.ltrs.digested.gff3  |egrep "^#"|egrep "###" -v >
Cobs.alpha.2.1.ltrs.digested.header
## add header to *.clustered.gff3
cat Cobs.alpha.2.1.ltrs.digested.header Cobs.alpha.2.1.ltrs.clustered.gff3 >
Cobs.alpha.2.1.ltrs.clustered.header.gff3
## Run gt to remove LTR retrotransposon candidates without any domain hit
(e.g. fragmented elements)
gt select -rule_files filter_full_domain_set.lua -- <
Cobs.alpha.2.1.ltrs.clustered.header.gff3 >
Cobs.alpha.2.1.ltrs.clustered.filtered.gff3
```

Select element to screen

In this example, we selected a single element to use for primer design from `Cobs.alpha.2.1.ltrs.clustered.filtered.gff3`. We use `LTR_retrotransposon798`. It is annotated as follows in the `gff3` file:

```
###
scaffold2   LTRharvest  repeat_region   900670  906001  .   -   .
ID=repeat_region798;ltrfam=ltrfam_8
scaffold2   LTRharvest  target_site_duplication 900670  900673  .   -   .
Parent=repeat_region798
scaffold2   LTRharvest  LTR_retrotransposon 900674  905997  .   -   .
ID=LTR_retrotransposon798;Parent=repeat_region798;ltr_similarity=98.43;seq_nu
mber=8
scaffold2   LTRharvest  long_terminal_repeat    900674  901183  .   -   .
Parent=LTR_retrotransposon798
scaffold2   LTRdigest   protein_match   901364  902258  0   -   .
Parent=LTR_retrotransposon798;reading_frame=1;name=_INT_csrn1_NA_INT_NA;clid=
3
scaffold2   LTRdigest   protein_match   901364  902258  0   -   .
Parent=LTR_retrotransposon798;reading_frame=1;name=INT_csrn1;clid=3
scaffold2   LTRdigest   protein_match   901613  902204  6.6e-21 -   .
Parent=LTR_retrotransposon798;reading_frame=1;name=_INT_osvaldo_NA_INT_NA;cli
d=3
scaffold2   LTRdigest   protein_match   901613  902204  6.9e-21 -   .
Parent=LTR_retrotransposon798;reading_frame=1;name=INT_osvaldo;clid=3
scaffold2   LTRdigest   protein_match   901616  902258  3.1e-32 -   .
Parent=LTR_retrotransposon798;reading_frame=1;name=_INT_epsilonretroviridae_N
A_INT_NA;clid=2
```

```
scaffold2   LTRdigest   protein_match   901616  902258  3.2e-32 -   .
Parent=LTR_retrotransposon798;reading_frame=1;name=INT_epsilonretroviridae;cl
id=2
[...]
scaffold2   LTRdigest   protein_match   903965  904199  2.1e-23 -   .
Parent=LTR_retrotransposon798;reading_frame=1;name=AP_csrn1;clid=0
scaffold2   LTRdigest   protein_match   903965  904199  2.2e-23 -   .
Parent=LTR_retrotransposon798;reading_frame=1;name=_AP_csrn1_NA_AP_NA;clid=0
scaffold2   LTRdigest   protein_match   904735  905188  4.2e-45 -   .
Parent=LTR_retrotransposon798;reading_frame=2;name=GAG_csrn1;clid=0
scaffold2   LTRdigest   protein_match   904735  905188  4.2e-45 -   .
Parent=LTR_retrotransposon798;reading_frame=2;name=_GAG_csrn1_NA_GAG_NA;clid=
0
scaffold2   LTRharvest  long_terminal_repeat    905490  905997  .   -   .
Parent=LTR_retrotransposon798;clid=68
scaffold2   LTRharvest  target_site_duplication 905998  906001  .   -   .
Parent=repeat_region798
```

The `ltr_similarity=98.43` indicates that the two long-terminal repeats of this elements are nearly identical (>98 %) and, overall, the annotation suggests that this is a complete and potentially active LTR element in the genome.

Once we selected an element to use for primer design, we extract the LTR sequences.

For tne primer design, we will only need the following two files to start: The `LTRharvest` annotation (`Cobs.alpha.2.1.ltrs.clustered.filtered.gff3`) amd the genome fasta file (`Cobs.alpha.2.1.fa`).

Extract fasta sequence of long_terminal_repeat for a given element

First, we extract the LTR sequence of `LTR_retrotransposon798`.

```
# define which element to target
elementID=LTR_retrotransposon798

# extract long_terminal_repeat sequence from both ends of the transposon with
bedtools
cat Cobs.alpha.2.1.ltrs.clustered.filtered.gff3|perl -pe
's/(.+)$/$1;/g'|egrep "$elementID;"|awk '{if ($3=="long_terminal_repeat")
print $0}' >  $elementID.ltrs.gff
bedtools getfasta -bed $elementID.ltrs.gff -fi Cobs.alpha.2.1.fa >
$elementID.ltrs.fa

# align both long_terminal_repeats with mafft
mafft --ep 0 --genafpair --maxiterate 1000  --clustalout $elementID.ltrs.fa >
$elementID.ltrs.aln
```

The alignment of the two LTR sequences looks as follows:

```
CLUSTAL format alignment by MAFFT E-INS-i (v7.490)


scaffold2:90067 gtttaggcttttcactagttcgcgggataggaaacgcgcaggatgtgtagcgaaataacc
scaffold2:90548 -tttaggcttttcactagttcgcgggataggaaacgcgcaggatgtgtagcgaaataacc
                 ***********************************************************

scaffold2:90067 caagagagaaataaagttaataaagtatatttcaaaccaatataaaaagggaagagacga
scaffold2:90548 caagagagagataaagttaataaagtatatttcaaaccaatataaaaagggaagagacga
                *********.**************************************************

scaffold2:90067 tgacaaagtaaaaaggaatacgaagactcgatacaaaactaattggaaaccgtcgcgtgc
scaffold2:90548 tgacaaagtaaaaaggaatacgaagactcgatacaaaactaattggaaaccgtcgcgttc
                ********************************************************** *

scaffold2:90067 gcgcggaacgacttcgagtgctggactgacgctccgacgcgtcgccagcaaggaagcgca
scaffold2:90548 gcgcggaacgatttcgagtgctggactgacgctccgacgcgtcgccagcaaagaagcgca
                ***********.***************************************.********

scaffold2:90067 agaggaagtctctttgtggcttatcagccctttgttaaagacgggacttcccggtgcatc
scaffold2:90548 agaggaagtctctttgtggcttatcagccctttgttaaagacgggacttcccggtgcatc
                ************************************************************

scaffold2:90067 atgcgagctgatgcaatgctgcgtcagcggaagctgacgaaaaggcttctagaaggtgta
scaffold2:90548 atgcgagctgatgcaatgctgcgtcagcggaagctgacgaaaaggcttctagaaggtgta
                ************************************************************

scaffold2:90067 aggtccagggtcccgttgccaagccaggctgttgctaagcaagggagaagatagtgcgcg
scaffold2:90548 aggtccagggtcccgttgccaagccaggctgttgctaggcaagggagaagatagtgcgcg
                *************************************.**********************

scaffold2:90067 gcactatcaattgcttgcgccgacggccggccgcaggtgataccggtaccgtcatctgcc
scaffold2:90548 gcactatcaattgcttgcgccgacggccggccgcaggtgataccggtaccgtcatctgcc
                ************************************************************

scaffold2:90067 gaggtgcgggcaaatccgcacccacatcca
scaffold2:90548 gaggtgcgggcaaatccgcacccacatac-
                *************************** *

# retrieve consensus sequence with cons
# http://emboss.sourceforge.net/apps/cvs/emboss/apps/cons.html

cons $elementID.ltrs.aln $elementID.ltrs.consensus.fa -name $elementID

# create 20 bp long regions (i.e. dummy primers) for this
long_terminal_repeat with samtools faidx
samtools faidx $elementID.ltrs.consensus.fa

# loop over the entire length of the consensus LTR sequence and extract 20 bp
```

```
long primers
## create coordinates for each primer
length=$(cut -f 2 $elementID.ltrs.consensus.fa.fai)
for i in $(seq 1 $((length-20)));
do
  start=$((1+$i))
  end=$((20+$i))
  echo $elementID:$start-$end
done > $elementID.regions

## extract 20 bp chunks of the LTR consensus of this element
samtools faidx $elementID.ltrs.consensus.fa -r $elementID.regions >
$elementID.regions.fa
```

This produces a files with all possible 20 bp primers along the consesnus sequence:

```
>LTR_retrotransposon798:2-21
TTTAGGCTTTTCACTAGTTC
>LTR_retrotransposon798:3-22
TTAGGCTTTTCACTAGTTCG
>LTR_retrotransposon798:4-23
TAGGCTTTTCACTAGTTCGC
>LTR_retrotransposon798:5-24
AGGCTTTTCACTAGTTCGCG
>LTR_retrotransposon798:6-25
GGCTTTTCACTAGTTCGCGG
>LTR_retrotransposon798:7-26
GCTTTTCACTAGTTCGCGGG
>LTR_retrotransposon798:8-27
CTTTTCACTAGTTCGCGGGA
>LTR_retrotransposon798:9-28
TTTTCACTAGTTCGCGGGAT
>LTR_retrotransposon798:10-29
TTTCACTAGTTCGCGGGATA
>LTR_retrotransposon798:11-30
TTCACTAGTTCGCGGGATAG
>LTR_retrotransposon798:12-31
TCACTAGTTCGCGGGATAGG
[...]
```

Next, we search these primers against the reference genome using `blastn`

```
# setup the genome as a local blast database
makeblastdb -dbtype nucl -in Cobs.alpha.2.1.fa
# blast primers of 20bp against the genome using blastn and produce a tabular
output
blastn -db Cobs.alpha.2.1.fa -num_threads 4 -query  $elementID.regions.fa -
word_size 7 -dust no -evalue 5 -outfmt 6  -perc_identity 100 -qcov_hsp_perc
100 >  $elementID.regions.bls
```

```
# create bed file from blast results
cat $elementID.regions.bls |awk '{print
$2"\t"$9"\t"$10"\t"$1";"$3";"$4";"$5";"$6";"$7";"$8";"$11";"$12}' >
$elementID.regions.bed

# check how many perfect hits each dummy primer produces
cut -f 1 $elementID.regions.bls |sort|uniq -c|perl -pe 's/^ +([0-9]+)
/$1\t/g'|sort -nr -k 1,1 > $elementID.regions.list
```

You can inspect the output of the last commands to see how many predicted primer binding sites you find in the assembly. `less $elementID.regions.list`

We see that several of the primers bind up to 49 times in the genome.

> 49 LTR_retrotransposon798:320-339 49 LTR_retrotransposon798:319-338 49 LTR_retrotransposon798:318-337 49 LTR_retrotransposon798:254-273 49 LTR_retrotransposon798:253-272 48 LTR_retrotransposon798:252-271 47 LTR_retrotransposon798:256-275 47 LTR_retrotransposon798:255-274 46 LTR_retrotransposon798:323-342 46 LTR_retrotransposon798:321-340

Select one primer and check overlap of primer binding sites with annotated TEs

We decide to use primer `LTR_retrotransposon798:407-426`, which has the following 40 binding sites in the genome:

```
LTR_retrotransposon798:407-426  scaffold49      100.000 20      0       0
1       20      404464  404483  0.002   38.1
LTR_retrotransposon798:407-426  scaffold72      100.000 20      0       0
1       20      32955   32936   0.002   38.1
LTR_retrotransposon798:407-426  scaffold72      100.000 20      0       0
1       20      37789   37770   0.002   38.1
LTR_retrotransposon798:407-426  scaffold25      100.000 20      0       0
1       20      6216236 6216217 0.002   38.1
LTR_retrotransposon798:407-426  scaffold25      100.000 20      0       0
1       20      6262906 6262887 0.002   38.1
LTR_retrotransposon798:407-426  scaffold50      100.000 20      0       0
1       20      6223    6242    0.002   38.1
LTR_retrotransposon798:407-426  scaffold50      100.000 20      0       0
1       20      11036   11055   0.002   38.1
LTR_retrotransposon798:407-426  scaffold43      100.000 20      0       0
1       20      136400  136419  0.002   38.1
LTR_retrotransposon798:407-426  scaffold43      100.000 20      0       0
1       20      141239  141258  0.002   38.1
LTR_retrotransposon798:407-426  scaffold41      100.000 20      0       0
1       20      103130  103111  0.002   38.1
LTR_retrotransposon798:407-426  scaffold41      100.000 20      0       0
1       20      107955  107936  0.002   38.1
LTR_retrotransposon798:407-426  scaffold41      100.000 20      0       0
1       20      241123  241142  0.002   38.1
LTR_retrotransposon798:407-426  scaffold41      100.000 20      0       0
```

```
1	20	251905	251886	0.002	38.1
LTR_retrotransposon798:407-426	scaffold39	100.000	20	0	0
1	20	310860	310879	0.002	38.1
LTR_retrotransposon798:407-426	scaffold38	100.000	20	0	0
1	20	188003	188022	0.002	38.1
LTR_retrotransposon798:407-426	scaffold30	100.000	20	0	0
1	20	230708	230727	0.002	38.1
LTR_retrotransposon798:407-426	scaffold30	100.000	20	0	0
1	20	235521	235540	0.002	38.1
LTR_retrotransposon798:407-426	scaffold23	100.000	20	0	0
1	20	3940443	3940424	0.002	38.1
LTR_retrotransposon798:407-426	scaffold19	100.000	20	0	0
1	20	433573	433554	0.002	38.1
LTR_retrotransposon798:407-426	scaffold19	100.000	20	0	0
1	20	438403	438384	0.002	38.1
LTR_retrotransposon798:407-426	scaffold17	100.000	20	0	0
1	20	140824	140805	0.002	38.1
LTR_retrotransposon798:407-426	scaffold17	100.000	20	0	0
1	20	145677	145658	0.002	38.1
LTR_retrotransposon798:407-426	scaffold17	100.000	20	0	0
1	20	601472	601453	0.002	38.1
LTR_retrotransposon798:407-426	scaffold16	100.000	20	0	0
1	20	460735	460716	0.002	38.1
LTR_retrotransposon798:407-426	scaffold16	100.000	20	0	0
1	20	561620	561601	0.002	38.1
LTR_retrotransposon798:407-426	scaffold18	100.000	20	0	0
1	20	4548844	4548863	0.002	38.1
LTR_retrotransposon798:407-426	scaffold18	100.000	20	0	0
1	20	4553609	4553628	0.002	38.1
LTR_retrotransposon798:407-426	scaffold14	100.000	20	0	0
1	20	4663057	4663038	0.002	38.1
LTR_retrotransposon798:407-426	scaffold14	100.000	20	0	0
1	20	4667877	4667858	0.002	38.1
LTR_retrotransposon798:407-426	scaffold14	100.000	20	0	0
1	20	4988572	4988553	0.002	38.1
LTR_retrotransposon798:407-426	scaffold1	100.000	20	0	0
1	20	6272044	6272025	0.002	38.1
LTR_retrotransposon798:407-426	scaffold1	100.000	20	0	0
1	20	6563677	6563696	0.002	38.1
LTR_retrotransposon798:407-426	scaffold2	100.000	20	0	0
1	20	235421	235402	0.002	38.1
LTR_retrotransposon798:407-426	scaffold2	100.000	20	0	0
1	20	516594	516613	0.002	38.1
LTR_retrotransposon798:407-426	scaffold2	100.000	20	0	0
1	20	878679	878698	0.002	38.1
LTR_retrotransposon798:407-426	scaffold2	100.000	20	0	0
1	20	901080	901099	0.002	38.1
LTR_retrotransposon798:407-426	scaffold2	100.000	20	0	0
1	20	905895	905914	0.002	38.1
LTR_retrotransposon798:407-426	scaffold6	100.000	20	0	0
```

```
1       20      669465  669484  0.002   38.1
LTR_retrotransposon798:407-426  scaffold6       100.000 20      0       0
1       20      717825  717806  0.002   38.1
LTR_retrotransposon798:407-426  scaffold8       100.000 20      0       0
1       20      7411764 7411745 0.002   38.1
```

Next, we can check how many of these primer binding sites overlap with a predicted LTR sequence from the `LTRharvest` annotation.

```
primerSelect=LTR_retrotransposon798:407-426

# with which predicted LTRs from LTRharvest does the primer overlap?
cat $elementID.regions.bed|grep $primerSelect|awk -v OFS='\t' '{if ($2<$3)
{print $0} else {print $1,$3,$2,$4}}'| bedtools intersect -b - -a
Cobs.alpha.2.1.ltrs.clustered.filtered.gff3 -F 1|awk '{if
($3=="LTR_retrotransposon") print $0}'
```

This shows that there are 21 predicted LTRs that overlap with the primer binding site. This is promising to provide a good number of amplicons in the Transposon Display in the end.

```
scaffold14      LTRharvest      LTR_retrotransposon     4663039 4663057 .
+       .
ID=LTR_retrotransposon100;Parent=repeat_region100;ltr_similarity=99.42;seq_nu
mber=12
scaffold14      LTRharvest      LTR_retrotransposon     4667859 4667877 .
+       .
ID=LTR_retrotransposon100;Parent=repeat_region100;ltr_similarity=99.42;seq_nu
mber=12
scaffold18      LTRharvest      LTR_retrotransposon     4548845 4548863 .
-       .
ID=LTR_retrotransposon184;Parent=repeat_region184;ltr_similarity=97.56;seq_nu
mber=15
scaffold18      LTRharvest      LTR_retrotransposon     4553610 4553628 .
-       .
ID=LTR_retrotransposon184;Parent=repeat_region184;ltr_similarity=97.56;seq_nu
mber=15
scaffold16      LTRharvest      LTR_retrotransposon     460717  460735  .
+       .
ID=LTR_retrotransposon194;Parent=repeat_region194;ltr_similarity=95.63;seq_nu
mber=16
scaffold17      LTRharvest      LTR_retrotransposon     140806  140824  .
+       .
ID=LTR_retrotransposon222;Parent=repeat_region222;ltr_similarity=99.81;seq_nu
mber=17
scaffold17      LTRharvest      LTR_retrotransposon     145659  145677  .
+       .
ID=LTR_retrotransposon222;Parent=repeat_region222;ltr_similarity=99.81;seq_nu
mber=17
scaffold19      LTRharvest      LTR_retrotransposon     433555  433573  .
+       .
```

```
ID=LTR_retrotransposon271;Parent=repeat_region271;ltr_similarity=99.05;seq_nu
mber=18
scaffold19	LTRharvest	LTR_retrotransposon	438385	438403	.
+	.
ID=LTR_retrotransposon271;Parent=repeat_region271;ltr_similarity=99.05;seq_nu
mber=18
scaffold30	LTRharvest	LTR_retrotransposon	230709	230727	.
-	.
ID=LTR_retrotransposon437;Parent=repeat_region437;ltr_similarity=99.61;seq_nu
mber=28
scaffold30	LTRharvest	LTR_retrotransposon	235522	235540	.
-	.
ID=LTR_retrotransposon437;Parent=repeat_region437;ltr_similarity=99.61;seq_nu
mber=28
scaffold41	LTRharvest	LTR_retrotransposon	107937	107955	.
+	.
ID=LTR_retrotransposon542;Parent=repeat_region542;ltr_similarity=95.88;seq_nu
mber=34
scaffold43	LTRharvest	LTR_retrotransposon	136401	136419	.
-	.
ID=LTR_retrotransposon546;Parent=repeat_region546;ltr_similarity=99.25;seq_nu
mber=36
scaffold43	LTRharvest	LTR_retrotransposon	141240	141258	.
-	.
ID=LTR_retrotransposon546;Parent=repeat_region546;ltr_similarity=99.25;seq_nu
mber=36
scaffold50	LTRharvest	LTR_retrotransposon	6224	6242	.
-	.
ID=LTR_retrotransposon604;Parent=repeat_region604;ltr_similarity=98.82;seq_nu
mber=41
scaffold50	LTRharvest	LTR_retrotransposon	11037	11055	.
-	.
ID=LTR_retrotransposon604;Parent=repeat_region604;ltr_similarity=98.82;seq_nu
mber=41
scaffold72	LTRharvest	LTR_retrotransposon	32937	32955	.
+	.
ID=LTR_retrotransposon713;Parent=repeat_region713;ltr_similarity=99.24;seq_nu
mber=66
scaffold72	LTRharvest	LTR_retrotransposon	37771	37789	.
+	.
ID=LTR_retrotransposon713;Parent=repeat_region713;ltr_similarity=99.24;seq_nu
mber=66
scaffold2	LTRharvest	LTR_retrotransposon	878680	878698	.
-	.
ID=LTR_retrotransposon797;Parent=repeat_region797;ltr_similarity=88.72;seq_nu
mber=8
scaffold2	LTRharvest	LTR_retrotransposon	901081	901099	.
-	.
ID=LTR_retrotransposon798;Parent=repeat_region798;ltr_similarity=98.43;seq_nu
mber=8
```

```
scaffold2	LTRharvest	LTR_retrotransposon	905896	905914	.
-	.
ID=LTR_retrotransposon798;Parent=repeat_region798;ltr_similarity=98.43;seq_nu
mber=8
```

In silico PCR

We next use https://github.com/egonozer/in_silico_pcr for *in-silico* PCRs, to test how the amplicons for our selected primer and a selected restriction site would look like.

We first extract the primer we want to test.

```
# retrieve primer sequence for selected primer
samtools faidx $elementID.regions.fa  $primerSelect > $primerSelect.fa
```

The sequence of the primer is: >>LTR_retrotransposon798.regions.fa:407-426
GAAGATAGTGCGCGGCACTA

Check GC and Tm

We can check GC content and melting temperature using the `R` library `TmCalculator` (https://github.com/JunhuiLi1017/TmCalculator) to make sure that the primer is feasible to use in a PCR.

```
RScript -e
"TmCalculator::Tm_GC('${primSeq}');TmCalculator::GC('${primSeq}');"
```

Next, we have to decide on which restriction enzyme to use. Here, we can adjust the number and size of amplicons we expect to get. We decided to use EcoR1, a rare cutting restriction enzyme.

Test a given primer with a restriction site (e.g. EcoR1) as reverse primer

Now, we will run an in silico PCR to explore how many amplicons we can roughly expect in our Transposon Display with primer `LTR_retrotransposon798:407-426` and EcoR1.

EcoR1 restriction site *CTTAAG*

The restriction site for EcoR1 is `CTTAAG`. This will not be the actual reverse primer in our Transposon Display, but the amplicons will in fact range from the forward primer binding site to the next restriction site as we will digest the DNA and ligate the reverse adapter to the DNA breaks. Thus, we can use the restriction site here in the in silico PCR as the reverse primer to simulate the PCR amplification we expect in the Transposon Display PCR.

```
# define primer name as a variable
primName=$(echo $primerSelect|perl -pe 's/:|-/./g')
# define primer sequence as a variable
primSeq=$(samtools faidx $elementID.regions.fa  $primerSelect|sed 1d)
# define reverse complement sequence as a variable
primSeqRevComp=$(samtools faidx $elementID.regions.fa  $primerSelect|seqkit
seq -r -p|sed 1d)
```

```
# git clone https://github.com/egonozer/in_silico_pcr.git
# Run in silico PCR with forward primer
perl in_silico_pcr/in_silico_PCR.pl -s Cobs.alpha.2.1.fa -a $primSeq -b
CTTAAG -l 5000 > $primName.fw.EcoR1.txt 2> $primName.fw.EcoR1.fa &
# Run in silico PCR with forward primer in reverse complement
perl in_silico_pcr/in_silico_PCR.pl -s Cobs.alpha.2.1.fa -a $primSeqRevComp -
b CTTAAG -l 5000 > $primName.rv.EcoR1.txt 2> $primName.rv.EcoR1.fa
grep -c "amp_" $primName.fw.EcoR1.txt
grep -c "amp_" $primName.rv.EcoR1.txt
```

The *in silico* PCR produces 32 amplicons of less than 5 kb size, which is a reasonable number of amplicons for our Transposon Display: > 17 amplicons of < 5 kb expected for primer > 15 amplicons of < 5 kb expected for reverse complement primer

Inspect *in silico* amplicons

We used the IGV genome browser (https://software.broadinstitute.org/software/igv/) to visually inspect the *in silico* predicted primer binding sites, the predicted amplicons and their expected overlap with known TE annotations. In IGV, load the genome `Cobs.alpha.2.1.fa` and add TE annotations:

- Cobs.alpha.2.1.ltrs.clustered.filtered.gff3
- primer binding sites (see below)
- *in silico* amplicons (see below)

1. Retrieve binding sites for primer based on blast results

   ```
   cat $elementID.regions.bed|grep $primerSelect >
   $primerSelect.regions.bed # load in IGV
   ```

2. Retrieve in silico PCR amplicons (e.g. `$primName.fw.EcoR1.bed`) bash cat $primName.fw.EcoR1.txt|awk -F $'\t' 'BEGIN {OFS = FS} {print $2,$3-1,$3+$4-1,$1";length="$4}' |sed 1d > $primName.fw.EcoR1.bed cat $primName.rv.EcoR1.txt|awk -F $'\t' 'BEGIN {OFS = FS} {print $2,$3-1,$3+$4-1,$1";length="$4}' |sed 1d > $primName.rv.EcoR1.bed

The IGV screenshot below shows two *in silico* predicted amplicons on the reverse strand (in red) for the TE family LTR_retrotransposon798, as annotated by LTR harvest. Small green annotations indicate the primer binding sites for the TE specific primer `LTR_retrotransposon798:407-426`. Note that only amplicon `amp_13` has the desired structure, covering the insertion site of the LTR element. Amplicons emerging from this configuration are suitable to reveal LTR element insertion polymorphisms between samples. In contrast, amplicon `amp_14` is contained entirely inside the LTR element, extending from the 5' end of the terminal repeat region into the LTR element. Amplification of this locus *in situ* is not suitable to reveal polymorphic insertions. However, such an amplicon will be featured prominently in the Transposon Display as a high intensity band in the agarose gel, as the same identical amplicon can be produced by any identical LTR element found in the genome. E.g. if the LTR element we target here has 10 identical copies

in the genome, then each of these 10 loci will function as a template for an amplicon identical to `amp_14` visualized here.

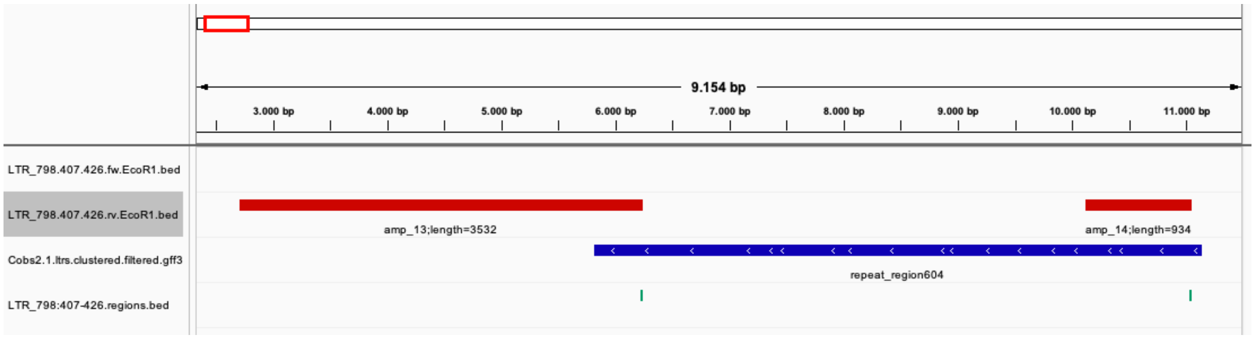

Supplement: bpae050_Supplementary_Data [file bpae050_supplementary_data.zip › SupplMaterial.pdf]
